# Temperature-driven shifts in microbial reactions and community structure in bentonite under Fe(III)- and sulfate-reducing conditions: implications for deep geological repository performance

**DOI:** 10.1128/aem.02005-25

**Published:** 2026-04-17

**Authors:** Kanghyun Park, Yidan Zhang, Yun Seo Jang, Sang-Ho Lee, Jang-Soon Kwon, Jeongdae Im, Man Jae Kwon

**Affiliations:** 1 Department of Earth and Environmental Sciences, Korea University, Seoul, South Korea; 2 Korea Atomic Energy Research Institute (KAERI) 65405, Daejeon, South Korea; 3 Department of Civil Engineering, Kansas State University 5308, Manhattan, Kansas, USA; Colorado School of Mines, Golden, Colorado, USA

**Keywords:** radioactive waste disposal, deep geological repositories, subsurface microbiology, thermophiles, anaerobic metabolism

## Abstract

Bentonite is a promising buffer material for the engineered barrier system in deep geological repositories (DGRs) of spent nuclear fuel. Beyond its physical barrier function, bentonite harbors microbial communities that can mediate biogeochemical interactions with surrounding materials, potentially impacting long-term repository stability. Over repository timescales, these microbes encounter a wide temperature range from radionuclide decay heat. In this study, we evaluated temperature effects on microbial processes and community dynamics under Fe(III)- and sulfate-reducing conditions. Microcosms of a Ca-type bentonite with lactate as an electron donor were incubated at 18–70°C. Results revealed a clear temperature-dependent shift in microbial reaction pathways. Low temperatures (18–30°C) exhibited lactate utilization via fermentation with active microbial reduction of both Fe(III) and sulfate. In contrast, high temperatures (40–70°C) favored lactate metabolism through incomplete oxidation to acetate followed by rapid sulfate reduction and comparatively lower Fe(III) reduction. Microbial community analysis identified common, metabolically flexible taxa across all temperatures, as well as temperature-dependent development of distinct sulfate- reducing bacteria, implying the ability of indigenous WRK microbes to survive and adapt to elevated temperatures. Our findings provide novel insights into bentonite microbial dynamics under repository-relevant conditions, advancing understanding of microbial interactions critical for long-term stability and safety of DGRs.

**IMPORTANCE:**

After the construction of the spent nuclear fuel (SNF) repository, the initial ambient environment of the bentonite barrier is subject to residual radioactive decay heat. Subsequently, the elevated temperature conditions within the repository (40–70°C) can persist for >1,000 years. Furthermore, a temperature gradient can be generated from the center of SNF storage area to the surface of the host rock, as the boundary of the SNF is at natural groundwater temperature (18–30°C). Given that SNF repositories must remain stable for over 100,000 years, understanding how microbial activity responds to these temperature conditions is critical for long-term repository safety.

## INTRODUCTION

The long-term management of high-level radioactive wastes including spent nuclear fuel (SNF) generated from nuclear power energy production is a significant global challenge. To accomplish this task, deep geological repositories (DGRs) are recognized as a primary solution in many countries for efficient and safe disposal of high-level radioactive wastes (1). A DGR, located deep underground within stable geological formation, can isolate these waste materials over geological timescales. Common concept of DGR implements a multi-barrier system composed of both the engineered barrier system and natural barriers (i.e., host rocks) to secure high-level radioactive wastes encapsulated in metal containers composed of steel or copper (2). Bentonite clays are widely adopted as buffer and backfill material in engineered barrier systems of several DGRs due to their physicochemical properties such as high swelling capacity and adsorption ability (3–5).

Microbes can be widespread in diverse subsurface environments, including DGR sites, and overall populations range 2–6 × 10^29^ cells, with microbial density varying according to multiple factors such as host rock lithology and redox conditions (6–8). Indigenous microbes in bentonite can be introduced to the DGR system as the main exogenous microbial source (9, 10). These microorganisms within engineered barrier systems can mediate various biogeochemical reactions interacting with surrounding materials both directly and indirectly. Metal containers are the first and most important barrier in engineered barrier systems that prevent the release of radionuclides to outer systems (11). Therefore, microbially influenced corrosion of metal containers made of steel or copper may be critical for stability of DGRs (12). Among various microbes, sulfate reducing bacteria (SRB) are identified as the most significant groups for microbially influenced corrosion in DGR environment. After repository closure, oxygen is gradually consumed through abiotic corrosion, microbial respiration, and reactions with host-rock minerals, bentonite, and concrete, driving the transition to an anoxic environment typical of DGR systems (13–15). Under anoxic conditions, SRB derive energy by reducing sulfate or other oxidized sulfur compounds, producing sulfide—a critical corrosive compound that accelerates metal canister degradation. Iron-reducing bacteria (IRB) may also contribute to corrosion by removing protective layers on metal surfaces through the reduction of insoluble ferric compounds (16).

Microorganisms within engineered barrier system can interact with the minerals causing weathering, dissolution, and formation of secondary minerals (17). Especially for bentonites, changes in physicochemical properties induced by microbial interactions could affect the performance as buffer material and further compromise barrier stability. However, under realistic DGR conditions—including high-density bentonite, saline groundwater, elevated temperature, low organic carbon and water content, and anoxia—microbial activity is expected to remain largely dormant. For example, a 5-year *in situ* experiment with highly compacted MX-80 bentonite (1.25–1.50 g/cm^3^) exposed to granitic groundwater showed that although SRB were present in outer layers, they were almost undetectable in the bentonite core, and microbial abundance remained similar to the initial state (18). Elevated temperature further suppresses microbial survival, with heating to 90–150°C significantly reducing viability under repository-relevant conditions (19). Overall, swelling pressure, limited pore size, and low water activity restrict microbial metabolism and preserve bentonite stability (20, 21). Nonetheless, localized interfaces or lower-density regions may create temporary microenvironments that permit limited microbial activity.

Several studies have demonstrated that microorganisms can survive and, under certain favorable circumstances, exhibit limited metabolic activity. For instance, Bengtsson and Pedersen (9, 22) and Martinez-Moreno et al. (20) reported the persistence and, in some cases, measurable activity of microbes within compacted bentonite. Bengtsson and Pedersen (22) further noted that spore-forming microorganisms can remain viable over extended timescales, and Maanoja et al. (23) suggested that even small amounts of organic matter in bentonite may sustain long-term sulfate-reducing activity. Over repository timescales, structural or physicochemical changes—such as micro-fissure development, alteration at bentonite interfaces, or interaction with infiltrating groundwater—may locally reduce compaction and create transient niches for microbial reactivation. Investigating such potential processes under controlled laboratory conditions, as simulated in this study, is, therefore, critical for evaluating all plausible scenarios in DGR safety assessments.

After DGR construction, surrounding systems could be subjected to a wide range of temperature conditions. In general, temperature in DGR and engineered barrier system increase mainly due to radioactive decay of SNF and minorly by hydration of cementitious materials depending on disposal concept (24). Landolt et al. (25) conceptually suggested changes in temperature-relative humidity at the canister surface from the emplacement up to one million years into four phases: (1) initial (aerobic) dry-out phase, (2) aerobic unsaturated phase, (3) anaerobic unsaturated phase, and (4) long-term cool, anoxic phase. Canister-surface temperatures may rise to ~140°C within the first 5–10 years (phase 1) before gradually decreasing to 40°C over tens of thousands of years. Especially at phase 4, the bentonite layer surrounding the canister can be fully saturated (100% relative humidity) with all remaining oxygen consumed by corrosion during the earlier phases.

Temperature is one of the most critical factors affecting microbial activity and survival. Generally, 122°C is currently recognized as the upper limit for prokaryotic activity in the subsurface environment, with some archaeal methanogens and iron reducers can survive up to this temperature (26, 27). Activity of sulfate reducers has also been identified in hydrothermal environments reaching 90–110°C (28, 29). Microbes can withstand extreme conditions by forming spores or endospores, and many SRB species can survive long periods at elevated temperatures through sporulation (9, 30). However, these upper limits and reported microbial activities were mainly upon specific microbes adapted and evolved to live in extreme temperature environments (e.g., hydrothermal environment). Thus, it is uncertain whether indigenous microbes originally present in the bentonite within the DGR environment could survive and mediate microbial reactions under such elevated temperatures. For instance, activities of indigenous microbes in Barbarian bentonite were confirmed with incubation at 60°C, suggesting the existence of thermophilic bacteria in bentonite (31). Since other bentonites, including the most widely studied Wyoming bentonite, commonly contain spore-forming SRB such as *Desulfosporosinus*, *Desulfitobacterium*, and *Desulfotomaculum* (31, 32), their potential activity under elevated temperatures must be evaluated before bentonite is used as a barrier material.

To evaluate the potential short-term impacts of microbial activity on WRK bentonite, a Ca-type bentonite commonly tested in South Korea as an engineered barrier material for nuclear waste disposal, conditions representing the most adverse scenario for a DGR were simulated. Although the repository environment is expected to be largely inhospitable to microbial life (e.g., due to elevated temperature, high radioactivity, and strong compaction), localized niches may still allow limited microbial activity. Understanding these possibilities is crucial for assessing long-term radionuclide retention and migration. Therefore, laboratory microcosms containing bentonite slurries amended with lactate as an electron donor as well as Fe(III)/sulfate as electron acceptors were used to investigate temperature-dependent microbial processes. In our previous study, we confirmed the ability of indigenous microbes in WRK bentonite to reduce Fe(III) (ferrihydrite) and sulfate using lactate as the primary electron donor under two different temperature conditions of 18°C and 50°C (33). Microbial reaction pathways differed markedly between temperatures: both acetate and propionate were produced at 18°C, whereas only acetate formed at 50°C. Fe(III) reduction was greater at 18°C, while sulfate reduction dominated at 50°C. Microbial community analysis showed distinct communities at each temperature and identified different taxa responsible for Fe(III) and sulfate reduction. Furthermore, the indigenous microbial community in bentonite included heat-resistant taxa capable of Fe(III) and sulfate reduction, processes that may adversely affect bentonite properties but also promote radionuclide immobilization, thereby influencing the overall stability of DGR systems in complex ways.

Based on these findings, we expanded our questions upon the impact of the temperature on microbial activities of WRK bentonite indigenous microbes. What is the threshold temperature inducing distinct changes in microbial reaction pathways? How will indigenous microbial communities shift as a response of elevating temperatures? To answer these questions, we conducted a batch microcosm experiment under more detailed temperature conditions ranging from 18°C to 70°C. Specific aims of this study were to (i) assess the microbial reaction pathways for lactate utilization and corresponding Fe(III) and sulfate reaction and (ii) investigate the shift in microbial community and identify temperature-specific development of functional taxa from indigenous microbes in WRK bentonite along temperatures. Lactate was used as a model electron donor because it is a central intermediate in subsurface carbon cycling (34, 35) and enables controlled examination of Fe(III)- and sulfate-reducing processes. Although lactate itself is unlikely to occur in bentonite porewaters, more complex or naturally derived organic substrates would likely promote similar pathways but at lower rates due to reduced bioavailability.

## RESULTS

Although microbial reaction rates varied among replicates at different temperatures, the reactions were grouped into two distinct types based on the metabolic fate of lactate. At 18°C and 30°C, both acetate and propionate were produced during lactate consumption, indicating fermentation. In contrast, only acetate was detected at 40°C, 50°C, 60°C, and 70°C, suggesting a shift in metabolic pathway toward incomplete oxidation. Therefore, we classified the results into low-temperature (18°C and 30°C) and high-temperature (40°C, 50°C, 60°C, and 70°C) conditions.

### Aqueous chemistry and microbial communities at low temperature conditions (18°C and 30°C)

**Fig 1:**
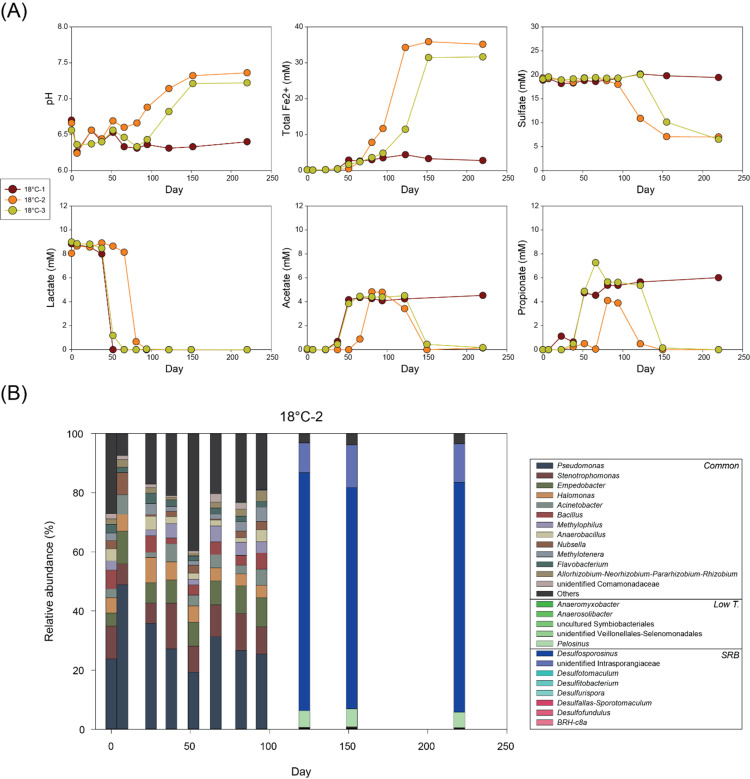
Changes in (**A**) pH and concentrations of total Fe(II), sulfate, lactate, acetate, and propionate and (**B**) microbial community composition at genus level over time in 18°C-2 bottle.

At 18°C, the time required for complete lactate consumption slightly differed among replicate bottles (Fig. 1A). Lactate was consumed within 66 days in bottles 18°C-1 and 18°C-2, and within 95 days in 18°C-3. During this period, concentrations of acetate and propionate increased with a minor increase in Fe(II) concentrations. Following lactate depletion, bottle 18°C-1 showed no further geochemical changes, with Fe(II), sulfate, acetate, and propionate remaining stable until day 220. In contrast, the other two bottles (18°C-2 and 18°C-3) showed rapid consumption of acetate and propionate, accompanied by increased Fe(II), decreased sulfate, and rising pH.

Microbial community analysis in bottle 18°C-2 showed little change until day 95 (Fig. 1B), dominated by *Pseudomonas*, *Stenotrophomonas*, *Empedobacter*, *Halomonas*, *Acinetobacter*, *Bacillus*, *Methylophilus*, *Anaerobacillus*, *Nubsella*, *Methylotenera*, *Flavobacterium*, *Allorhizobium-Neorhizobium-Pararhizobium-Rhizobium*, and unidentified Comamonadaceae. These taxa were also dominant in high-temperature and autoclaved control treatments and were, therefore, defined as common taxa. The shared genera corresponded to identical amplicon sequence variants (ASVs) detected across treatments rather than distinct ASVs within the same genus. Extraction blanks and PCR negative controls yielded negligible read counts and did not contain these dominant ASVs.

A dramatic shift in microbial community was observed along with decreasing sulfate concentrations (days 122–220), with *Desulfosporosinus* dominating (78% average relative abundance), followed by unidentified members of family Intrasporangiaceae (12%) and *Pelosinus* (6%).

**Fig 2:**
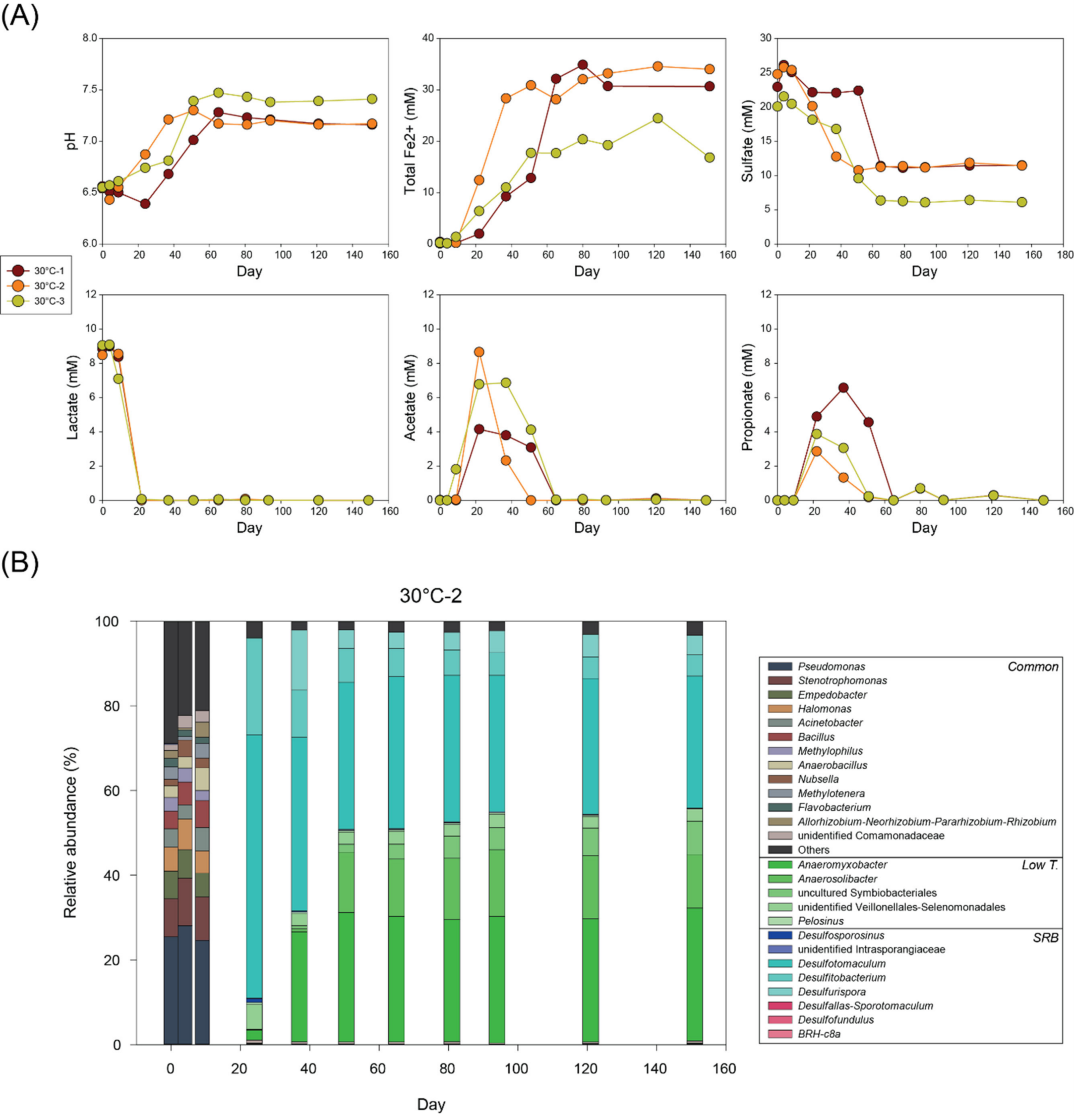
Changes in (**A**) pH and concentrations of total Fe(II), sulfate, lactate, acetate, and propionate and (**B**) microbial community composition at genus level over time in 30°C-2 bottle.

At 30°C, geochemical changes occurred more rapidly than at 18°C (Fig. 2A). Lactate was depleted within 24 days in all replicate bottles, with concurrent production of acetate and propionate. The acetate-to-propionate ratio varied across replicates, with bottles 30°C-1 and −2 showing higher acetate concentrations than propionate. Not only a slight increase in Fe(II) concentrations as at 18°C but also a minor decrease in sulfate concentrations was observed during this phase. Following lactate depletion, acetate and propionate were consumed with increasing Fe(II) concentration and decreasing sulfate. After depletion of both acetate and propionate at day 65, no further geochemical changes were observed.

Microbial communities in bottle 30°C-2 exhibited similar early composition as at 18°C (Fig. 2B), dominated by common taxa (e.g., *Pseudomonas* [26%], *Stenotrophomonas* [10.1%], *Empedobacter* [6.3%], *Halomonas* [6.1%], *Bacillus* [5.3%], and *Acinetobacter* [4.4%]). A significant shift of microbial community was also observed at 30°C with rapid decrease in sulfate concentrations. *Desulfotomaculum* and *Desulfitobacterium* exhibited significant dominance by day 24 with relative abundances of 62.1% and 22.9%, respectively. With slightly decreased abundances of *Desulfotomaculum* (41%) and *Desulfitobacterium* (11.2%), *Anaeromyxobacter* (26%) and *Desulfurispora* (14.2%) emerged by day 37. From day 51, microbial communities maintained stability until the end of the experiment (day 151). During this period, *Desulfotomaculum* (33.4%) and *Anaeromyxobacter* (29.9%) were observed dominantly followed by decreased *Desulfitobacterium* (6%) and *Desulfurispora* (4.6%), with appearance of *Anaerosolibacter* (14.3%) uncultured Symbiobacteriales members (5.7%).

### Aqueous chemistry and microbial communities at high temperature conditions (40°C, 50°C, 60°C, and 70°C)

**Fig 3:**
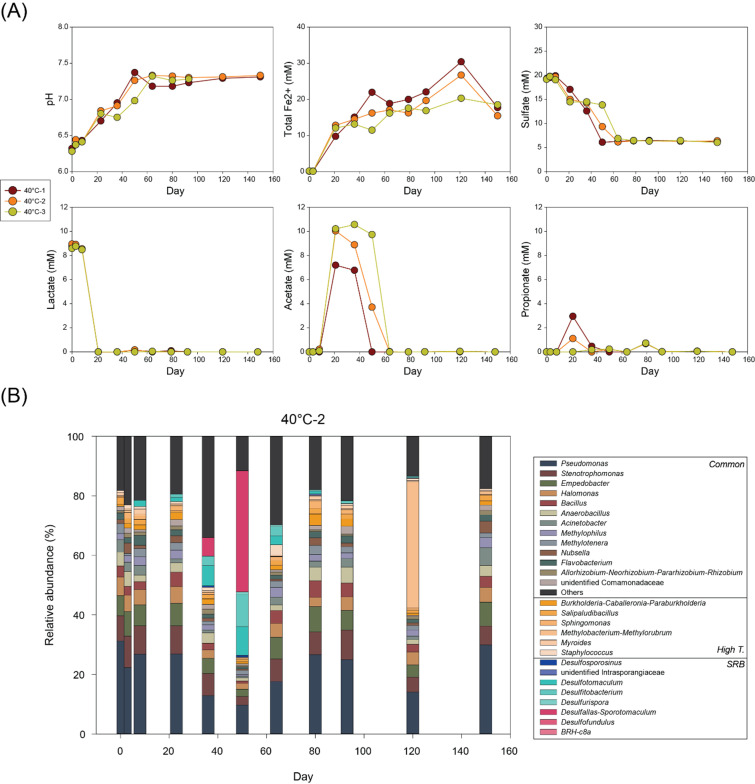
Changes in (**A**) pH and concentrations of total Fe(II), sulfate, lactate, acetate, and propionate and (**B**) microbial community composition at genus level over time in 40°C-2 bottle.

**Fig 4:**
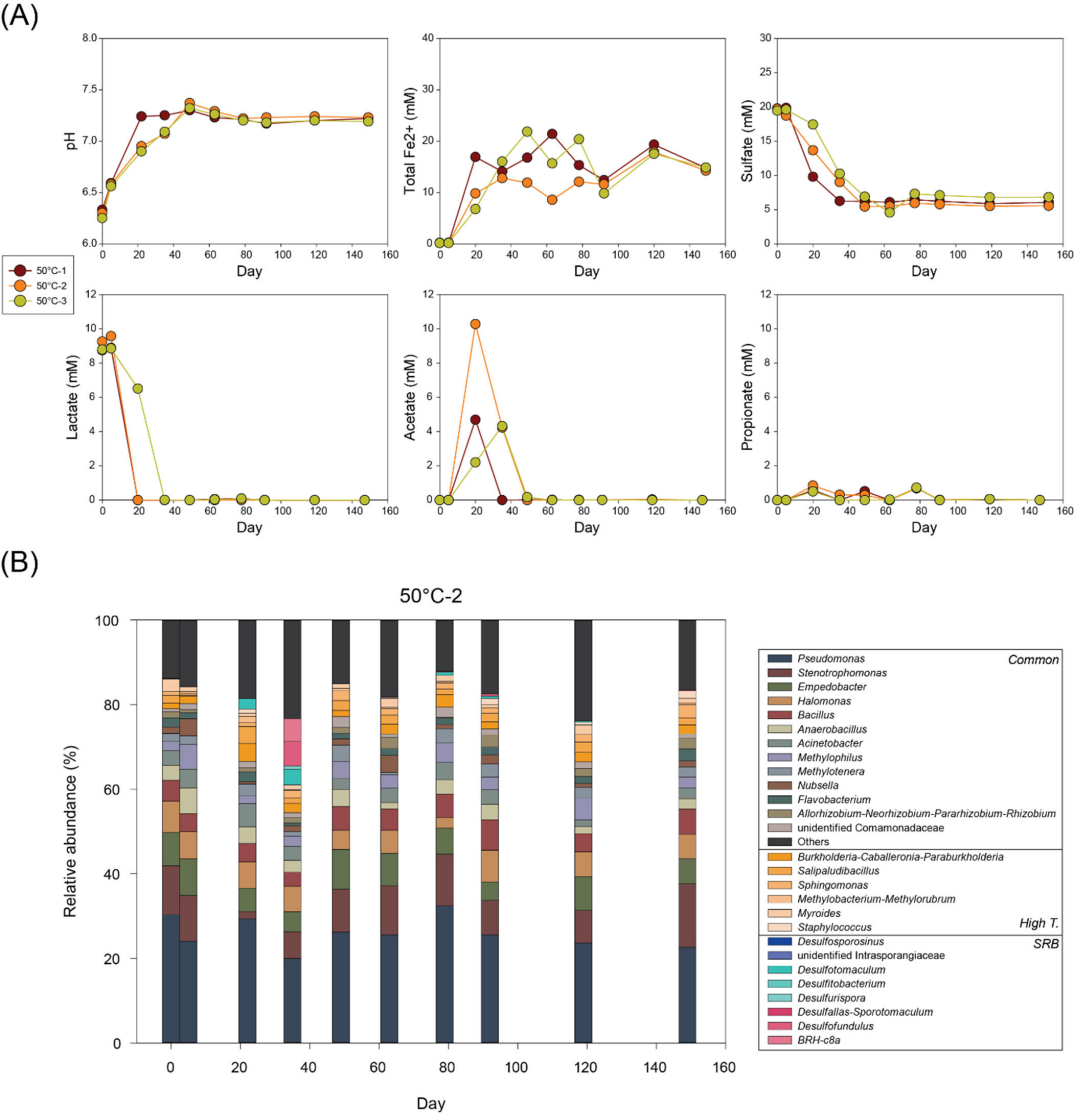
Changes in (**A**) pH and concentrations of total Fe(II), sulfate, lactate, acetate, and propionate and (**B**) microbial community composition at genus level over time in 50°C-2 bottle.

**Fig 5:**
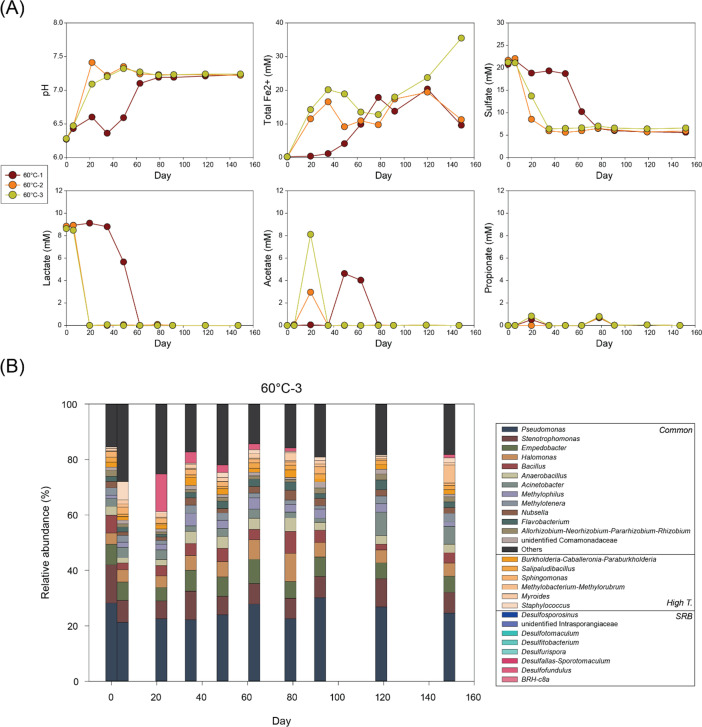
Changes in (**A**) pH and concentrations of total Fe(II), sulfate, lactate, acetate, and propionate and (**B**) microbial community composition at genus level over time in 60°C-3 bottle.

**Fig 6:**
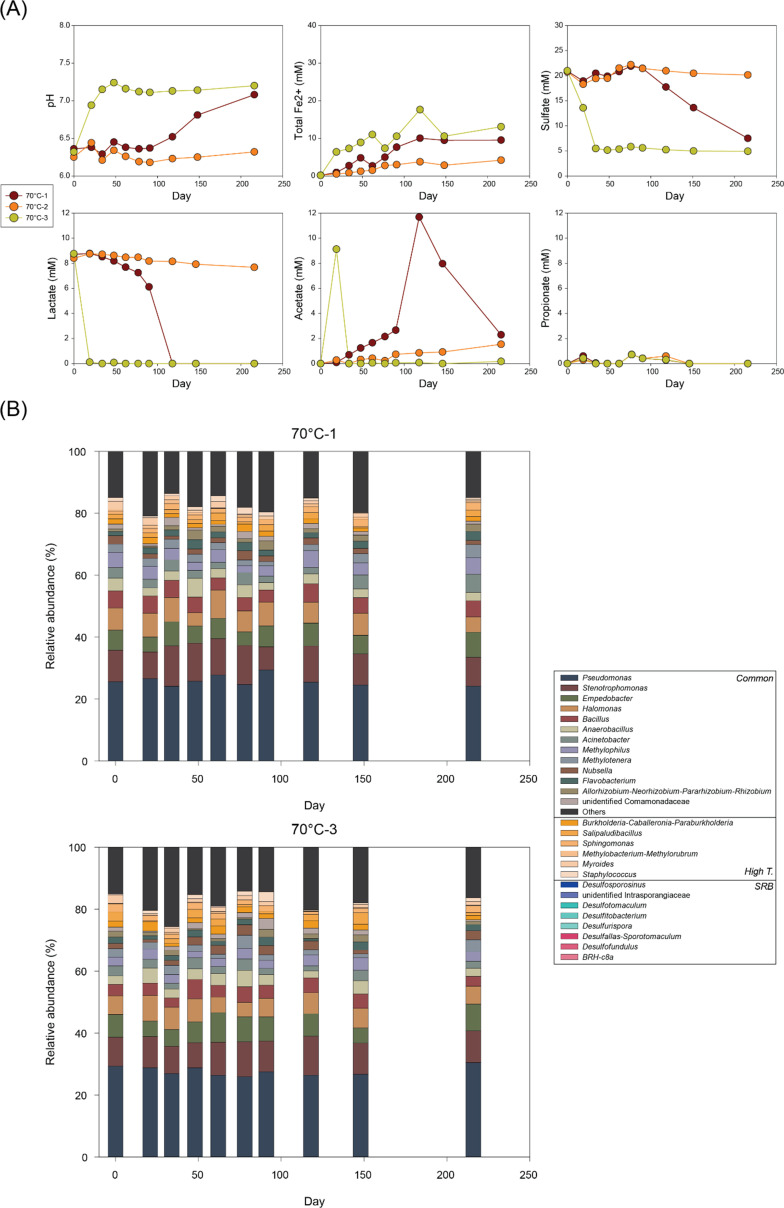
Changes in (**A**) pH and concentrations of total Fe(II), sulfate, lactate, acetate, and propionate and (**B**) microbial community composition at genus level over time in 70°C-1 and 70°C-3 bottles.

High temperature conditions (40–70°C) generally revealed similar microbial reactions though reaction rates and microbial activities varied with temperature and across replicate bottles (Fig. 3A, 4A, 5A and 6A). At 40°C and 50°C, lactate was depleted within 22 days in most bottles, accompanied by increases in acetate and Fe(II) concentrations and decreases in sulfate concentrations (Fig. 3A and 4A). However, acetate concentrations varied considerably among bottles due to immediate acetate consumption following its formation. After lactate depletion and acetate formation, both acetate and sulfate were rapidly decreased, while Fe(II) concentrations relatively remained stable.

At 60°C, microbial activity in bottles 60°C-2 and 60°C-3 was comparable to that observed at 40°C and 50°C (Fig. 5A). Lactate was depleted within 22 days with increases in acetate and Fe(II) and decreases in sulfate. In contrast, 60°C-1 showed a delayed onset of microbial activity, with lactate consumption starting only after day 49. After initiation, reaction pathways and rates were similar to the other two bottles.

At 70°C, microbial activity appeared even more discretely along replicate bottles (Fig. 6A). In 70°C-3, microbial reactions occurred at a rate comparable to lower temperatures. In contrast, 70°C-1 showed slower reactions. Lactate decreased slowly from day 21 to day 91 accompanied by acetate production and increase in Fe(II) and rapidly depleted by day 118 with decreasing sulfate concentrations. Subsequently, the decrease in sulfate coupled with acetate consumption continued until the end of the experiment. Moreover, 70°C-2 revealed the slowest activity, with less than 2 mM of lactate consumed by the end of the experiment, resulting in equivalent formation of acetate and limited changes in Fe(II) and sulfate concentrations.

Microbial communities at high temperatures were generally dominated by common taxa that were also found at low temperatures (Fig. 3B, 4B, 5B and 6B). Additionally, several genera including *Burkholderia-Caballeronia-Paraburkholderia*, *Salipaludibacillus*, *Sphingomonas, Methylobacterium-Methylorubrum*, *Myroides*, and *Staphylococcus* were identified. Changes in microbial communities were also observed throughout high temperatures during decreases in sulfate concentrations. However, these changes occurred temporarily, reverting to original composition with common taxa thereafter.

At 40°C (bottle 40°C-2), *Desulfotomaculum* increased from 6.8% at day 36 to a peak of 9.7% by day 50 and then decreased to 2.9% by day 64 (Fig. 3B). *Desulfitobacterium* followed a similar trend, increasing from 3.2% to 10.9%, then declining to 3.1%. *Desulfallas-Sporotomaculum* emerged as a predominant genus increasing from 6.2% (day 36) to 40.7% (day 50), before disappearing by day 64.

From 50°C, changes in microbial communities appeared relatively insignificant compared to previous temperature conditions (Fig. 4B). However, *Desulfotomaculum* emerged in 50°C-2, increasing from 2.4% (day 22) to 3.6% (day 35). By day 35, *Desulfofundulus* and *BRH-c8a* also appeared with relative abundances of 5.8% and 5.4%, respectively.

At 60°C (60°C-3), *Desulfofundulus* appeared abundantly by day 22 with 13.5% of relative abundance and gradually decreased to 1.2% by day 79 (Fig. 5B). At 70°C, no significant community shifts were observed in either bottle 70°C-1 or 70°C-3, regardless of microbial reaction rates (Fig. 6B).

### Aqueous chemistry and microbial community changes in control conditions

Geochemical changes due to microbial activity in the control conditions varied even between replicate bottles at the same temperature (Fig. S1 to S3). In the 18°C, 30°C, and 40°C controls, one of the two bottles exhibited no microbial activity until the end of the experiment (day 220), with no changes in lactate concentration (Fig. S1 and S2A). At 60°C, both bottles showed similar microbial reactions but with significantly different reaction rates (Fig. S3A). In contrast, the replicates at 50°C showed consistent reaction trends and rates (Fig. 1B), while neither of 70°C control bottles showed any indication of microbial activity throughout the experiment (Fig. 2B).

**Fig 7:**
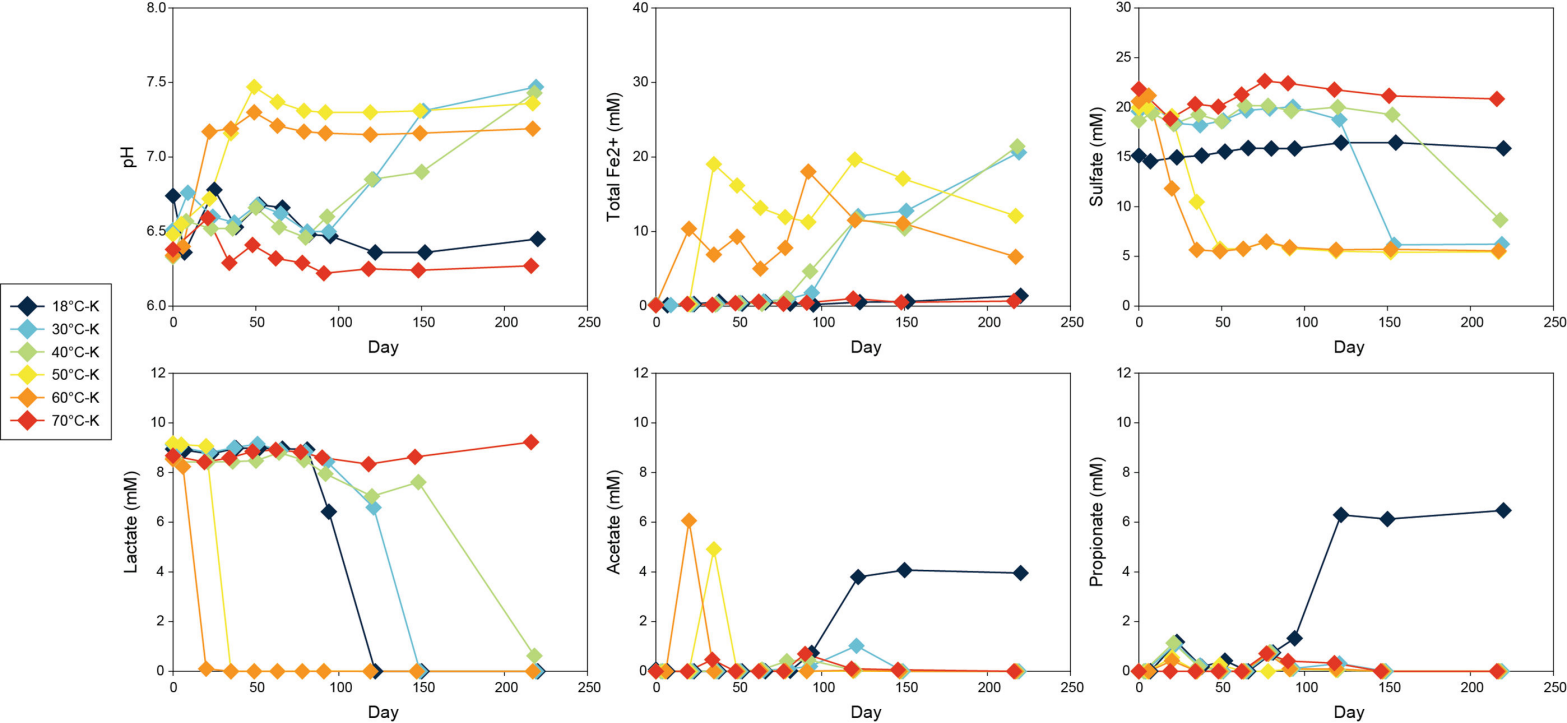
Changes in pH and concentrations of total Fe(II), sulfate, lactate, acetate, and propionate in representative kill control microcosms incubated at 18–70°C. Data reflect microbial activity and geochemical trends in sterilized bentonite under varied thermal conditions.

To assess microbial reactions and community changes under control conditions, we chose one representative bottle from each temperature in which microbial activity was observed (Fig. 7). In the 18°C control (18°C-K-1), lactate decreased between day 82 and day 122, accompanied by the formation of acetate and propionate. These organic acids remained stable until day 220 without significant changes in total Fe(II) and sulfate concentrations. In the 30°C to 60°C controls, geochemical changes progressed similar to those described for high temperature conditions in the previous section, only acetate formed after lactate consumption accompanied by increasing Fe(II) concentrations and decreasing sulfate concentrations.

Microbial community compositions remained relatively stable in the 18°C (18°C-K-2) and 70°C (70°C-K-2) controls and were mainly composed of common taxa and microbes abundant in high temperature conditions (Fig. S4). However, the 30°C (30°C-K-1) and 40°C (40°C-K-2) controls revealed significant shifts in microbial communities associated at the end of the experiment. In the 30°C control, *BRH-c8a* dominated the microbial communities at both day 152 and 220, with relative abundances of 92.1% and 82.7%, respectively. In the 40°C control, *Desulfurispora* became dominant (48.1%) at the last time point (day 218), with the presence of uncultured members within order Desulfitobacteriales (4.4%). In the 50°C (50°C-K-1) and 60°C (60°C-K-1 and -2) controls, microbial community changes were less dramatic than at 30°C and 40°C controls but still depicted clear responses to decreasing sulfate concentrations. *Desulfofundulus* was identified in the 50°C control at day 35 (4.2%) and in the 60°C control (60°C-K-2) at day 22 (3.4%).

## DISCUSSION

It should be noted that, despite efforts to homogenize the WRK bentonite, the use of natural clay inevitably introduces variability in the initial microbial community composition. This natural heterogeneity likely contributed to some inconsistencies observed in microbial activity and geochemical responses among replicate bottles. Rather than being attributed to experimental or analytical errors, these differences reflect the inherent biological complexity (e.g., possibly arising from minor differences in initial community composition or microenvironmental conditions) and unpredictability of engineered barrier systems under repository-relevant conditions. Importantly, such variability may influence microbial activity rates and reaction pathways—especially under varying temperature conditions—even when initially sterilized bentonite is used. This finding highlights the need to account for microbial heterogeneity in future assessments of barrier stability and long-term safety.

### Geochemistry and microbial community dynamics at low temperature conditions (18°C and 30°C)

Aqueous geochemistry results at 18°C clearly depicted fermentation as the primary metabolic pathway for lactate utilization. This pattern aligns with our previous findings (33). However, the observed acetate:propionate was close to 1:1, differing from the theoretical 3:1:2 molar ratio of lactate-to-acetate-to-propionate expected from lactate fermentation (equation 1) (36).


(1) $$3CH_{3}CHOHCOO^{-}\to CH_{3}COO^{-}+2CH_{3}CH_{2}COO^{-}+HCO_{3}^{-}+H^{+}$$

This deviation suggests an additional acetate-generating process may have occurred during lactate consumption. A slight increase in Fe(II) concentrations during this phase implies that incomplete oxidation of lactate to acetate coupled with Fe(III) reduction may have been a minor concurrent pathway (equation 2) (37).


(2) $$CH_{3}CHOHCOO^{-}+4Fe(OH{)}_{3}(s)+7H^{+}\to CH_{3}COO^{-}+4Fe^{2+}+HCO_{3}^{-}+10H_{2}O$$

Thus, our results indicate that while fermentation was the dominant pathway at 18°C, a minor contribution from incomplete lactate oxidation coupled with Fe(III) reduction likely contributed secondarily. After lactate consumption, two bottles (18°C-2 and 18°C-3) indicated Fe(III) and sulfate reduction. Due to the simultaneous nature of these changes, direct attribution of secondary electron donor (acetate and propionate) consumption to either Fe(III) or sulfate reduction was not straightforward. Nonetheless, based on prior studies, we infer that complete oxidation of acetate contributed to Fe(III) reduction (equation 3) (38) and sulfate reduction (equation 4) (39), while propionate supported sulfate reduction (equation 5) (40).


(3) $$CH_{3}COO^{-}+8Fe(OH{)}_{3}(s)+15H^{+}\to 2HCO_{3}^{-}+8Fe^{2+}+20H_{2}O$$

(4) $$CH_{3}COO^{-}+SO4^{2-}+H^{+}\to 2HCO_{3}^{-}+H_{2}S$$

(5) $$4CH_{3}CH_{2}COO^{-}+7SO_{4}^{2-}\to 12HCO_{3}^{-}+7HS^{-}+H^{+}$$

Given that the observed sulfate reduction exceeded what could be accounted by propionate oxidation alone, we conclude that acetate served as the primary electron donor for sulfate reduction, with only a minor fraction used for Fe(III) reduction. Additionally, the increase in total Fe(II) exceeded the expected levels from acetate oxidation, suggesting that abiotic Fe(III) reduction by biogenic sulfide also occurred (41), with concurrent pH increases attributed to proton (H^+^) consumption (equation 6).


(6) $$2Fe(OH{)}_{3}+H_{2}S+4H^{+}\to S^{0}+2Fe^{2+}+6H_{2}O$$

Microbial communities at 18°C (18°C-2) mainly consisted of common taxa during earlier microbial reaction phase until day 95 (Fig. 1B). Their persistence suggests that early-stage microbial reactions (i.e., lactate fermentation and minor incomplete oxidation with Fe(III) reduction) were likely mediated by this shared group. Detailed taxonomic and functional characteristics of common taxa are discussed in “Microbial community shift along temperature,” below.

A dramatic shift in the microbial community from day 122 resembled the sulfate-reducing phase and clearly implied the predominance of SRBs. *Desulfosporosinus* is well-known SRB capable of utilizing lactate, acetate, and propionate with sulfate reduction (42–44). While Intrasporangiaceae members are not typically classified as SRB, He et al. (45) identified an unidentified genus from this family under sulfate-rich condition. Therefore, while *Desulfosporosinus* likely played the dominant role in sulfate reduction, the concurrent increase of Intrasporangiaceae suggests a potential, yet unconfirmed, ecological association with sulfur cycling under these conditions.

At 30°C, lactate fermentation (equation 1) also appeared as initial and primary microbial metabolism as at 18°C. A slight increase in Fe(II) and a minor decrease in sulfate during this phase imply that incomplete lactate oxidation was coupled with both Fe(III) and sulfate reduction (equation 2 and 7) (46).


(7) $$4CH_{3}CHOHCOO^{-}+2SO_{4}^{2-}\to 4CH_{3}COO^{-}+4HCO_{3}^{-}+HS^{-}+H_{2}S$$

The subsequent reactions reflect sulfate reduction by complete oxidation of acetate (equation 4) and propionate (equation 5), accompanied by minor Fe(III) reduction with complete acetate oxidation (equation 3). Additional formation of Fe(II) might be associated with abiotic Fe(III) reduction by biogenic sulfide (equation 6).

In summary, microbial processes at 30°C were similar to those at 18°C but occurred faster and ended earlier due to the depletion of organic acids (lactate, acetate, and propionate). In addition, higher acetate production at 30°C than at 18°C suggests greater contribution from incomplete oxidation of lactate to acetate, emphasizing that this pathway becomes more favorable with increasing temperature even under low-temperature conditions.

Microbial communities at 30°C (30°C-2) were also dominated by common taxa during the early lactate-consuming phase until day 24. Therefore, this observation repeatedly implied that these common taxa mediated earlier reactions including lactate fermentation and minor incomplete oxidation with Fe(III) and sulfate reduction.

Following significant microbial community turnover also closely mirrored rapid sulfate reduction, with SRBs dominating the community as observed at 18°C. However, 30°C exhibited the presence of different SRBs compared to those that appeared at 18°C. These SRB and sulfur-metabolizing taxa are known for their metabolic flexibility. For example, *Desulfotomaculum*, *Desulfurispora*, and *Desulfitobacterium* can use sulfate and other sulfur compounds including thiosulfate, sulfite, and elemental sulfur (47–50). *Desulfitobacterium* is also known to have versatile metabolic potentials with various electron acceptors including nitrate, Fe(III), humic acids, and halogenated organic compounds (51).

### Geochemistry and microbial community dynamics at high temperature conditions (40°C, 50°C, 60°C, and 70°C)

Geochemical results from high temperature conditions consistently identified primary lactate utilization via incomplete oxidation to acetate accompanied with Fe(III) and sulfate reduction, followed by secondary acetate-based reduction. A minor amount of propionate was observed in 40°C-1, along with slightly lower acetate concentrations, implying that fermentation of lactate was still possible at 40°C. However, no other bottles at high temperatures showed propionate production, clearly indicating a metabolic shift from fermentation to incomplete oxidation beginning at 40°C.

Microbial reactions at 40°C and 50°C exhibited similar reaction pathways and rates. In bottles 40°C-2, -3, and 50°C-2, acetate concentrations approached those expected from full conversion of lactate, incomplete lactate oxidation coupled with Fe(III) reduction (equation 2) and sulfate reduction (equation 7). Rapid decreases in both acetate and sulfate after lactate depletion suggest complete oxidation of acetate with sulfate reduction (equation 4). The observed molecular ratio of acetate consumed to sulfate reduced was close to 1:1, consistent with the theoretical stoichiometry. Therefore, most acetate generated from lactate incomplete oxidation was likely oxidized completely during sulfate reduction. This pattern was consistent with trends in total Fe(II) which increased during lactate consumption and remained stable or variable after depletion of lactate. Compared to low temperature conditions, this suggests that abiotic Fe(III) reduction by biogenic sulfide was less significant at elevated temperatures.

Despite similar reaction pathways at lower temperatures, greater variability among triplicate microcosms was observed at elevated temperatures, including a delayed onset in one 60°C replicate and distinct differences in reaction rates at 70°C. These findings indicate that microbial activity and reaction kinetics of the bentonite indigenous community become increasingly sensitive and heterogeneous above 60°C rather than being completely inhibited.

The delayed onset of activity in one 60°C replicate suggests that elevated temperature may impose physiological stress on portions of the microbial community, potentially prolonging the lag phase prior to metabolic activation. However, because the other replicates exhibited measurable activity without comparable delay, the variability more likely reflects intrinsic biological heterogeneity within the WRK bentonite microcosms, including differences in initial community composition, dormancy state, or micro-scale physicochemical conditions.

Importantly, measurable sulfate reduction and organic acid transformation were still observed at 60–70°C, demonstrating that microbial processes persisted under these elevated temperatures. The reduced or delayed responses, therefore, likely reflect thermal suppression of metabolic rates rather than complete microbial inactivation. Many bentonite-associated microorganisms, particularly spore-forming taxa, are capable of entering dormant states under thermal stress and may remain viable during high-temperature phases. Thus, thermotolerant or dormant populations could sustain limited activity at elevated temperatures and potentially reactivate more rapidly if temperatures subsequently decline, as may occur in repository environments.

Observation on initial microbial communities at high temperatures implied that these common taxa shared throughout low- and high-temperature conditions mediate early microbial reactions involving incomplete lactate oxidation with Fe(III) and sulfate reduction. Following transient shifts in microbial communities were also indicated during the rapid sulfate reduction phase using acetate although dominant SRB differed by temperature. *Desulfotomaculum* and *Desulfitobacterium* are identified as dominating SRBs at 30°C and 40°C. *Desulfallas-Sporotomaculum* was dominant at 40°C but absent at low temperatures. *Desulfallas* is a novel SRB genus established through taxonomic revision of *Desulfotomaculum* (52).

SRB genera detected at 50°C were not observed at low temperatures or 40°C. *Desulfofundulus*, an SRB lineage within *Desulfotomaculum*, is composed predominantly of thermophiles (52–55). Although detailed functional or taxonomic characteristics of *BRH-c8a* within family *Desulfallas-Sporotomaculum* remain unclear, it shares sequence similarity with *Desulfotomaculum* found in deep subsurface (56), suggesting potential sulfate-reducing capability. As the temperature increases, transient changes with SRBs appeared relatively insignificant. *Desulfofundulus* was the only SRB detected at 60°C, while no SRB was identified at 70°C despite clear evidence of microbial sulfate reduction.

In summary, microbial communities at high temperatures indicated the presence and activity of SRB during sulfate reduction, primarily via acetate utilization. However, the dominant SRB taxa varied with temperature, and overall community turnover was less pronounced than at low temperatures, likely due to the reduced relative abundance of SRB under thermal stress. After sulfate reduction ceased, the detected community composition shifted toward dominance of the common ASVs observed across treatments. This pattern suggests a decline in temperature-enriched sulfate-reducing populations and a relative increase in background taxa, rather than wholesale community restructuring.

### Microbial reactions and community dynamics in control conditions

The results indicated the occurrence of microbial activity throughout control conditions except at 70°C (Fig. 7; Fig. S1 to S3). The 18°C control only exhibited lactate fermentation, while controls at 30°C to 60°C revealed similar reaction pathways to high temperature conditions with Fe(III) and sulfate reduction after lactate incomplete oxidation to acetate. Microbial community shifts along with sulfate reduction appeared only significant in 30°C and 40°C controls that dominating SRB of *BRH-c8a* and *Desulfurispora* were both identified in incubation conditions.

Despite three times of autoclaving, our results implied triple-autoclaving was insufficient to completely sterilize the bentonite. A similar result was presented in our previous study that 50°C autoclaved control amended with both Fe(III) and sulfate revealed fast microbial reactions as unsterilized incubation (33). It may be due to complex characteristics of clay minerals which limit complete sterilization of indigenous microbes in bentonite. A previous study has investigated the effectiveness of heat sterilization on bentonite and identified presences of heat-resistant microorganisms from bentonite powder even after nine cycles of autoclaving (57). Some SRB including *Desulfotomaculum* also has revealed ability to mediate sulfate reduction when exposed to high temperatures (40–60°C) after multiple autoclaving (58). Together, control condition results denote that thermotolerant microorganisms in bentonite remained viable even after sterilization via autoclaving and reactivated over time.

### Microbial community shift along temperature

**Fig 8:**
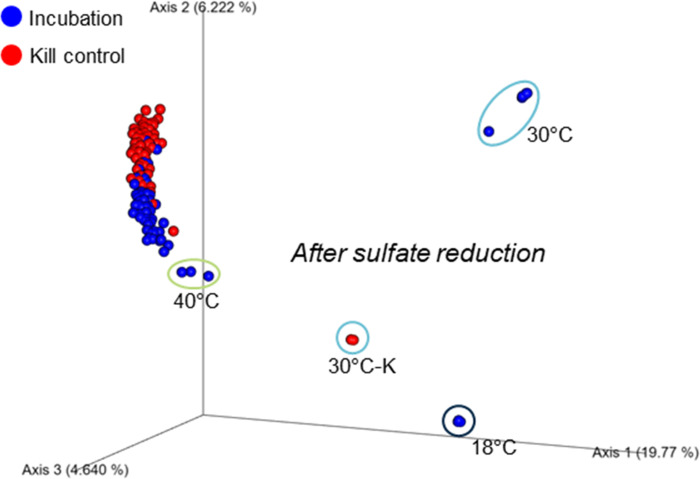
Principal coordinates analysis (PCoA) based on Bray-Curtis dissimilarity of microbial communities. Blue and red dots represent incubation and kill control samples, respectively. Distinct clustering of post sulfate-reduction communities is observed at 18°C, 30°C, and 30°C-K, indicating temperature-specific shifts.

Beta diversity was determined by Bray-Curtis distance principal coordinates analysis (PCoA) to assess dissimilarity of microbial communities (Fig. 8). The PCoA plot revealed a large cluster of microbial communities including most of incubation and kill control samples along temperatures. However, several distinct clusters emerged, particularly for samples collected after the sulfate reduction phase at 18°C, 30°C, and in the 30°C kill control. Communities during sulfate reduction at 40°C showed moderate separation from the main cluster. These observations are consistent with previous results indicating that microbial communities prior to sulfate reduction were largely composed of common taxa. As noted in earlier sections, low-temperature conditions (18°C and 30°C) led to significant community turnover during sulfate reduction, with the development of distinct SRB and sulfur-metabolizing taxa. In contrast, high-temperature communities showed only a moderate enrichment of SRB during sulfate reduction and subsequently reverted to their pre-reaction states. Thus, strong microbial community dissimilarity during sulfate reduction was apparent primarily at low temperatures, with only partial differentiation at 40°C.

Across all conditions, the following taxa were consistently identified as common microbial members: *Pseudomonas*, *Stenotrophomonas*, *Empedobacter*, *Halomonas*, *Acinetobacter*, *Bacillus*, *Methylophilus*, *Anaerobacillus*, *Nubsella*, *Methylotenera*, *Flavobacterium*, *Allorhizobium-Neorhizobium-Pararhizobium-Rhizobium*, and unidentified members of the Comamonadaceae. These taxa persisted throughout different temperatures and even under sterilization (kill control), suggesting their persistence as heat-resistant or spore-forming members.

Geochemical and diversity analyses collectively suggest that these core consortia mediate various microbial processes beyond sulfate reduction including lactate utilization via fermentation and incomplete oxidation and Fe(III) reduction. While *Pseudomonas* has not been definitively shown to ferment lactate to both acetate and propionate, it has been identified as a dominant taxon in sulfidogenic bioreactors using lactate alongside SRB and fermentative microbes (59). Some *Pseudomonas* species are also known to reduce Fe(III) (60, 61). Other common taxa exhibit notable metabolic and physiological traits. For instance, *Halomonas* is aerobic or facultatively anaerobic, with some species (e.g., *H. titanicae*) capable of dissimilatory Fe(III) reduction under anaerobic conditions (62). *Bacillus* is known for broad metabolic flexibility and prevalence in bentonite (63, 64). Some *Bacillus* species can reduce Fe(III) (65–67). Along with Fe(III) reducing potentials, *Bacillus* and *Anaerobacillus* also possess heat-resisting ability by forming endospores and spores (68, 69). *Methylophilus* and *Methylotenera,* as methylotrophic microbes, may help mitigate abiotic stress under high temperature conditions (70). Altogether, these common taxa not only mediate complex microbial reactions in our microcosm systems but also imply the ability to adapt in high temperatures or survive from sterilization.

To assess temperature-dependent selection of sulfate reducers, we compared the maximum relative abundances of SRB and sulfur metabolizers under different incubation temperatures and kill control conditions (Table 1). Identifying temperature-dependent shifts in dominant SRB taxa is essential to understand microbial adaptability in fluctuating thermal environments, especially in subsurface or engineered systems where temperature gradients may be pronounced. Distinct SRB taxa dominated at different temperatures. At 18°C, *Desulfosporosinus* was predominant, reaching a maximum relative abundance of 80.5%, while an unidentified member of the *Intrasporangiaceae* family also appeared (14.4%). With increased incubation temperature, *Desulfotomaculum* became dominant at 30°C (62.1%), decreasing at 40°C (9.6%) and 50°C (3.6%). *Desulfotobacterium* (22.9%) and *Desulfurispora* (14.2%) were also notable at 30°C. At 40°C, *Desulfallas–Sporotomaculum* emerged as a major taxon (40.7%), followed by *Desulfotobacterium* (10.9%) and *Desulfotomaculum* (9.6%). At 50°C, a shift was observed toward *Desulfofundulus* (5.8%) and *BRH-c8a* (5.4%). At 60°C, *Desulfofundulus* remained the only SRB detected (13.5%). No SRB were detected at 70°C, nor under kill control conditions, except some detection of *Desulfurispora* (48.1% at 40°C-K), *Desulfofundulus* (4.2%–3.4% at 50°C-K and 60°C-K), *BRH-c8a* (92.1% at 30°C-K), and uncultured *Desulfotobacteriales* (0.1%–4.4% at 30°C-K and 40°C-K). These results clearly demonstrate temperature-dependent selection and succession of SRB genera, reflecting ecological flexibility and niche specialization. The observed pattern aligns with previous findings that SRB can thrive across a wide temperature range (−5 to 75°C; 71), highlighting their adaptive potential under thermal stress.

**TABLE 1:** Maximum relative abundances (%) of SRB and sulfur metabolizers at genus level presented in each condition

	Incubation						Kill control (incubation after autoclaving)					
	18°C	30°C	40°C	50°C	60°C	70°C	18°C-K	30°C-K	40°C-K	50°C-K	60°C-K	70°C-K
*Desulfosporosinus*	80.5	0.9	0.6	–^*a*^	–	–	–	–	–	–	–	–
Unidentified intrasporangiaceae	14.4	0.2	0.5	–	–	–	–	–	–	–	–	–
*Desulfotomaculum*	–	62.1	9.6	3.6	–	–	–	–	–	–	–	–
*Desulfitobacterium*	–	22.9	10.9	–	–	–	–	–	–	–	–	–
*Desulfurispora*	–	14.2	0.8	0.8	–	–	–	–	48.1	–	–	–
*Desulfallas-Sporotomaculum*	–	–	40.7	–	–	–	–	–	–	–	–	–
*Desulfofundulus*	–	–	–	5.8	13.5	–	–	–	–	4.2	3.4	–
*BRH-c8a*	–	–	–	5.4	–	–	–	92.1		–	–	–
Uncultured desulfitobacteriales	–	–	–	–	–	–	–	0.1	4.4	–	–	–

^*a*^ – indicates less than 0.0%.

### Implications on deep geological repositories and limitation of the study

Our slurry experiments conducted with powdered WRK bentonite represent conditions far more permissive to microbial activity than those expected in compacted bentonite barriers within a deep geological repository. While dry density, water activity, pore-space restrictions, salinity, and temperature in engineered buffers impose strong limitations on microbial mobility and metabolism, the slurry setup was deliberately selected to simulate a “worst-case” environment that maximizes microbial growth and reactivity. By doing so, the study identifies microbial and biogeochemical processes that could theoretically occur if localized zones with elevated water availability, reduced compaction, or other deviations from ideal barrier conditions develop over long operational timescales. However, extrapolation of these findings to repository-relevant conditions must be made cautiously: the compacted bentonite matrix would significantly suppress microbial diffusion, nutrient transport, and metabolic rates, meaning that the magnitude and prevalence of reactions observed in slurries are unlikely to be replicated *in situ*.

Another limitation of this study is that ferrihydrite was used as a reactive Fe(III) phase to sensitively assess microbial Fe(III)-reducing activity. In natural or repository settings, more crystalline Fe(III) oxides (e.g., goethite, hematite, magnetite) would likely be reduced more slowly due to their lower surface reactivity (72, 73 and references therein) though similar microbial pathways may still operate under favorable conditions. Nonetheless, characterizing microbial behavior under non-limiting conditions provides a valuable baseline for understanding the upper bounds of potential microbial impacts and informs assessments of how rare micro-environments within the barrier could evolve.

Biogeochemical reactions were not observed uniformly across all three replicates, attributing the pattern solely to experimental error is not sufficient. Instead, the variability more likely reflects true biological heterogeneity among the microcosms, such as differences in initial microbial community composition, spore abundance, or micro-scale physicochemical conditions, rather than experimental noise. This interpretation is consistent with previous reports documenting variable lag phases among replicate microcosms arising from natural differences in microbial activation potential. In the context of repository safety assessment, the observed variability underscores the expectation that microbial processes in a deep geological repository will not occur uniformly throughout the engineered barrier but may instead manifest locally or episodically in microenvironments where conditions transiently favor metabolic activity. These findings reinforce the need for conservative evaluations that account for such heterogeneity, recognizing that even small differences in microbial seed populations or local geochemical conditions can lead to markedly different onset times and reaction rates.

Our findings also provide valuable insight into the temperature-dependent microbial processes that may occur within the bentonite buffer under the sub-100°C conditions expected throughout most of a DGR system. The observed decline—but not complete loss—of microbial activity at elevated temperatures suggests that certain heat-resistant or spore-forming microorganisms may remain viable in portions of the buffer that undergo moderate heating. However, an important limitation is that our experiments did not extend into the > 100°C range experienced directly at the canister surface, where long-term exposure may either sterilize the bentonite or select for only highly thermotolerant populations. Consequently, while our results help constrain microbial behavior across a realistic temperature window for the majority of the buffer, they cannot fully resolve the fate or potential reactivation of microorganisms in zones exposed to sustained temperatures approaching 140°C. Future work employing high-temperature, high-pressure reactors, other types of Fe(III) minerals, and long-term incubation is required to assess microbial survival, dormancy, and metabolic potential under truly repository-relevant thermal extremes.

### Conclusion

This study systematically investigated the impact of temperature on microbial reactions and community dynamics of WRK bentonite indigenous microbes under Fe(III)- and sulfate-reducing conditions across a wide thermal gradient (18–70°C) using controlled microcosm experiments. Results revealed a clear metabolic shift in lactate-utilizing pathways: low temperatures (18°C and 30°C) favored fermentation coupled with simultaneous Fe(III) and sulfate reduction, whereas high temperatures (40°C to 70°C) led to incomplete lactate oxidation to acetate, followed by rapid sulfate reduction and diminished Fe(III) reduction. These findings demonstrate that temperature not only modulates the dominant reaction mechanisms but also influences the extent and sequence of redox processes critical to engineered barrier performance.

Microbial community analysis highlighted two key patterns: (i) the persistence of metabolically versatile common taxa across all temperature conditions, such as *Pseudomonas*, *Stenotrophomonas*, *Halomonas*, *Bacillus*, and *Anaerobacillus*, which were capable of mediating fermentation, incomplete oxidation, and Fe(III) reduction—even after sterilization treatment and (ii) the emergence of temperature-specific SRB and sulfur-metabolizing taxa, including *Desulfosporosinus*, *Desulfotomaculum*, *Desulfurispora*, *Desulfallas*, and *Desulfofundulus*. The presence of these taxa reflects strong functional adaptation to thermal conditions and reveals the dynamic nature of bentonite-associated microbial communities.

Variations in microbial activity and geochemical responses among replicate bottles were observed despite efforts to homogenize the natural bentonite material. These inconsistencies reflect inherent heterogeneity in the initial microbial communities of natural clays. Such variability is representative of the complex and unpredictable nature of real-world engineered barrier systems. Even in sterilized bentonite, temperature gradients may stimulate microbial activity at different rates and extents, underscoring the importance of accounting for microbial diversity and spatial heterogeneity in safety assessments.

Together, our findings provide a comprehensive and temperature-resolved understanding of how indigenous bentonite microbes interact with key redox-active components under repository-relevant conditions. The insights gained here have direct implications for evaluating the long-term stability and safety of DGR systems, particularly in the context of thermal evolution following nuclear waste emplacement. The demonstrated resilience and functional plasticity of microbial consortia highlight the need to consider microbially driven geochemical processes in performance assessments of engineered barriers.

## MATERIALS AND METHODS

### Microcosm setup

The commercial powdered bentonite product (WRK bentonite) was obtained from Clariant (Korea) Ltd. (Pohang, South Korea). The mineralogical and geochemical characteristics of WRK bentonite (i.e., mineralogy, elemental composition, water content, and organic carbon contents) were determined in our previous study (Tables S1 and S2) (33).

The batch experiment was conducted using 160 mL serum bottles containing 125 mL of artificial groundwater (AGW) and 5 g of WRK bentonite as the indigenous microbial source, along with lactate as the electron donor and ferrihydrite and sulfate as the electron acceptors. To minimize contamination and ensure consistency, bentonite was carefully subsampled from the original commercial container by avoiding contact with surface layers and edges. The collected material was transferred into a sterilized autoclave jar and thoroughly mixed to promote homogeneity. For each replicate set of microcosms, bentonite was consistently taken from the same homogenized subsample jar to reduce experimental bias. In addition, small aliquots of roughly 1 g from this pre-mixed batch were dispensed into each experimental bottle in five sequential additions rather than adding the full five-gram portion at once. This stepwise distribution was intended to maintain consistency in the material allocated to each bottle and to minimize potential heterogeneity that could arise when withdrawing larger quantities from the mixed batch. All tools used for subsampling and transfer were sterilized using 70% ethanol and flame between each use. This procedure was implemented to minimize variability and ensure comparability across replicates while maintaining the native microbial community structure as much as possible.

The AGW was designed in the previous research to simulate the deep groundwater at the DB-3 borehole located in the Underground Research Tunnel (KURT) constructed by the Korea Atomic Energy Research Institute (KAERI, Daejeon, South Korea) (74). The AGW was composed of 1.41 mM of NaHCO_3_, 0.42 mM of NaF, 0.29 mM of Na_2_SiO_3_, 0.16 mM of CaCl_2_, 0.05 mM of MgSO_4_, 0.04 mM of NaCl, and 0.01 mM of KCl. The AGW was set with minimal ionic concentration and buffering capacity to serve as minimal basal media minimizing experimental bias. The AGW was sterilized by autoclaving at 121°C for 15 min. Ferrihydrite (Fh), a poorly crystalline Fe(III) oxyhydroxide, was synthesized modifying the previous methods (33, 75). In brief, 0.4 M of FeCl_3_ was dissolved in 200 mL of deionized (DI) water and then the pH was adjusted to 7.0 by titration with 10 M NaOH. After the titration, Fh in the form of slurry was centrifuged at 7,150 × *g* for 20 min, and supernatant was discarded. Then, Fh was resuspended in DI water and washed three times in the same condition. Finally, the Fh pellet was stored at 4°C to prevent further transformation. Stock solutions for lactate and sulfate were prepared separately using sodium lactate (NaC_3_H_5_O_3_) and sodium sulfate (Na_2_SO_4_). Stock solutions were made anoxic by 100% N_2_ purging and sterilized by autoclaving at 121°C for 15 min.

The AGW was transferred to each serum bottle and sterilized by autoclaving at 121°C for 15 min. Approximately 2 g of Fh was added aseptically to each bottle to achieve 32 mM of final Fe(III) concentration and pasteurized at 75°C for 30 min to sterilize Fh avoiding structural transformation. Pasteurized serum bottles containing AGW and Fh were opened aseptically, and 5 g of WRK bentonite powder was transferred to each serum bottle. Serum bottles were made anoxic by purging with N_2_:CO_2_ (90:10 vol%), capped with a butyl rubber stopper, and crimp sealed with an aluminum cap. Finally, lactate and sulfate stock solutions were amended aseptically using sterile syringes and needles to achieve 10 mM and 20 mM of final concentrations, respectively. For kill-control bottles, bentonite was sterilized by autoclaving three times before transfer. The bottles were incubated at temperatures of 18°C, 30°C, 40°C, 50°C, 60°C, and 70°C for 220 days in dark without agitation; for each temperature, incubation was performed in triplicate and duplicate for kill controls.

### Sampling and geochemical analysis

The sampling of microcosm bottles was conducted periodically to monitor the biogeochemical changes during the experiment. Subsamples were collected using sterile syringes and needles flushed with N_2_:CO_2_ (90:10 vol%) to maintain anoxic condition. Each microcosm bottle was vigorously shaken prior to sampling to ensure homogenization. Aliquots were filtered using a 0.22 μm nylon syringe filter to obtain samples for analysis of organic acids (lactate, acetate, and propionate), SO_4_^2−^, and aqueous Fe(II). Samples for organic acids and SO_4_^2−^ analysis were prepared by mixing 0.5 mL of filtrate with 0.5 mL of 20 mM H_2_SO_4_ and 0.5 mL of 30% isopropanol, respectively. Then samples for organic acids and SO_4_^2−^ analysis were stored at 4°C until analysis. For aqueous Fe(II) analysis, 0.2 mL of the filtrate was mixed with 0.2 mL of 1 M HCl avoiding exposure to the air. Aliquots of unfiltered samples were also taken for pH, total Fe(II), and microbial community analysis. Two of 1 mL aliquots of unfiltered samples were taken for pH measurements and microbial community analysis. Samples for microbial community analysis were centrifuged at 15,000 × *g* for 1.5 min to remove supernatant and stored at −20°C prior to DNA extraction. Samples for total Fe(II) analysis were prepared in the same way as those for aqueous Fe(II) analysis.

Measurement of pH and aqueous/total Fe(II) concentrations was conducted immediately after sampling. The pH was measured using an Orion Star multimeter and a pH microelectrode (Thermo Scientific, USA). The concentrations of aqueous and total Fe(II) in 0.5 M HCl extract were analyzed using the ferrozine assay method (76, 77) at a wavelength of 562 nm with DR3900 spectrophotometer (HACH, USA). SO_4_^2−^ concentration was determined with DIONEX ion chromatography (IC) with IonPac AS14 analytical column (Thermo Scientific, USA). Concentrations of organic acids (lactate, acetate, and propionate) were analyzed by 1260 Infinity high performance liquid chromatography (HPLC) system with a MetaCarb 87H Organic Acids Column and a refractive index detector (RID) (Agilent, USA).

### Microbial community analysis

The pellets for microbial community analysis preserved at −20°C were thawed at room temperature prior to DNA extraction. Total genomic DNA was extracted using DNeasy PowerSoil Pro kit (QIAGEN, Germany) following the manufacturer’s protocol. DNA concentrations in the extract solutions were quantified using a Qubit fluorometer (Invitrogen, USA). Amplicon library preparation and Illumina MiSeq paired-end (2 × 151 bp) sequencing of 16S rRNA gene were conducted by Argonne National Laboratory, USA. The V4 region of the 16S rRNA gene was amplified using 515F and 805R primer set for construction of MiSeq amplicon library (78). Each 25 µL PCR contains 9.5 µL of MO BIO PCR Water (Certified DNA-Free), 12.5 µL of QuantaBio’s AccuStart II PCR ToughMix (2× concentration, 1× final), 1 µL Golay barcode tagged Forward Primer (5 µM concentration, 200 pM final), 1 µL Reverse Primer (5 µM concentration, 200 pM final), and 1 µL of template DNA. The conditions for PCR are as follows: 94°C for 3 min to denature the DNA, with 35 cycles at 94°C for 45 s, 50°C for 60 s, and 72°C for 90 s with a final extension of 10 min at 72°C to ensure complete amplification. Raw sequence data were processed using QIIME2 version 2022.08 (79). Paired-end reads were demultiplexed and quality-filtered, and amplicon sequence variants (ASVs) were inferred using the DADA2 plugin, which performs denoising, merging of paired reads, and chimera removal. Taxonomy was assigned to ASVs using a Naïve Bayes classifier pre-trained on the SILVA database (version 138, 99% similarity) trimmed to the amplified region. Extraction blanks and PCR negative controls were sequenced alongside samples to assess potential contamination. ASVs detected in negative controls were removed from downstream analyses if present at comparable abundance levels. Following taxonomic assignment, sequences classified as chloroplast, mitochondria, eukaryotic, or unassigned were removed, retaining only bacterial and archaeal ASVs for analysis.

## Data Availability

All data generated or analyzed during this study are included in this published article and its supplemental material. The raw sequence data generated in this study have been deposited in the NCBI database under BioProject accession number PRJNA1435099.

## SUPPLEMENTAL MATERIAL

Supplemental material aem.02005-25-s0001.docx Tables S1 and S2; Fig. S1 to S4.

ASM does not own the copyrights to Supplemental Material that may be linked to, or accessed through, an article. The authors have granted ASM a non-exclusive, world-wide license to publish the Supplemental Material files. Please contact the corresponding author directly for reuse.
