# Supplementary material for: Temperature-driven shifts in microbial reactions and community structure in bentonite under Fe(III)- and sulfate-reducing conditions: implications for deep geological repository performance
Source: Appl Environ Microbiol. 2026 Apr 17;92(5):e02005-25. doi: 10.1128/aem.02005-25 (PMC13188865; doi:10.1128/aem.02005-25)
Supplement: Supplemental material — Tables S1 and S2; Fig. S1 to S4. [file aem.02005-25-s0001.docx]

Supplementary Information

**Temperature-Driven Shifts in Microbial Reactions and Community Structure in Bentonite Under Fe(III)- and Sulfate-Reducing Conditions: Implications for Deep Geological Repository Performance**

Kanghyun Park^1^, Yidan Zhang^1^, Yun Seo Jang^1^, Sang-Ho Lee^2^, Jang-Soon Kwon^2^, Jeongdae Im^3^, Man Jae Kwon^1*^

^1^Department of Earth and Environmental Sciences, Korea University, Seoul 02831, South Korea

^2^Korea Atomic Energy Research Institute (KAERI), Daejeon 34057, South Korea

^3^Department of Civil Engineering, Kansas State University, Manhattan, KS 66506, USA

**^*^Correspondence;
manjaekwon@korea.ac.kr**

**Table S1.** The mineralogy of powdered WRK bentonite used in this study (adopted from Park et al., 2024)

| **Minerals** | **wt%** |
| --- | --- |
| Montmorillonite | 75.8 |
| Plagioclase | 7.4 |
| Calcite | 4.7 |
| Quartz | 3.7 |
| Clinoptilolite | 3.3 |
| Cristobalite | 2.7 |
| K feldspar | 2.0 |
| Mica | 0.4 |

**Table S2.** The elemental composition of powdered WRK bentonite used in this study (adopted from Park et al., 2024)

| **Elements** | **wt%** |
| --- | --- |
| SiO_2_ | 64.3 ± 0.0^b^ |
| Al_2_O_3_ | 18.0 ± 0.0 |
| TiO_2_ | 0.5 ± 0.0 |
| Fe_2_O_3_ | 3.9 ± 0.0 |
| MnO | 0.1 ± 0.0 |
| MgO | 2.6 ± 0.0 |
| CaO | 2.8 ± 0.0 |
| Na_2_O | 0.3 ± 0.0 |
| K_2_O | 0.8 ± 0.0 |
| P_2_O_5_ | 0.1 ± 0.0 |
| LOI^a^ | 6.6 ± 0.0 |
| Total | 99.9 ± 0.0 |

^a^ LOI = Loss-on-ignition

^b^ Standard deviation


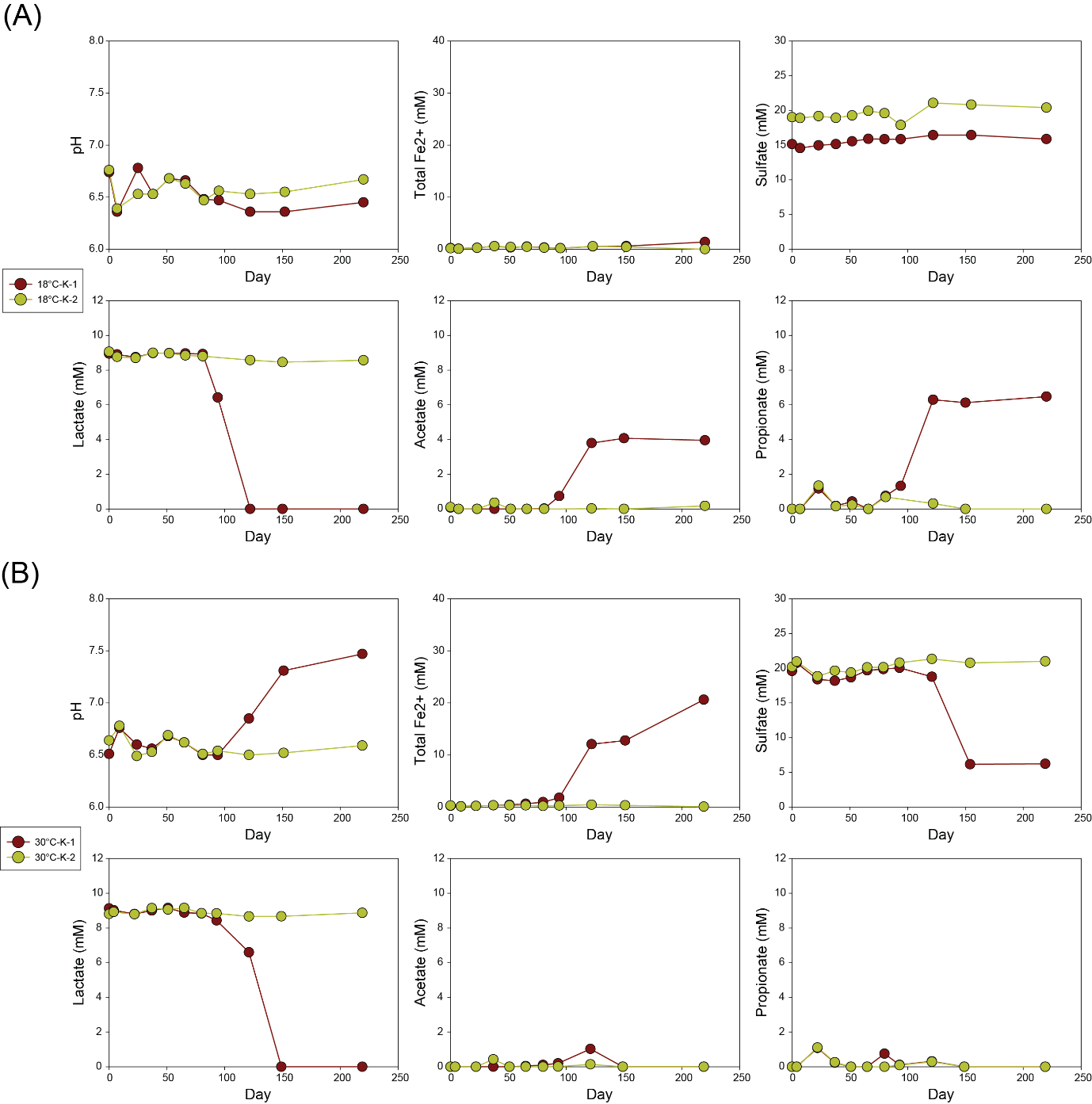


**Figure S1.** Changes in pH and concentrations of total Fe(II), sulfate, lactate, acetate, and propionate over time in kill control incubations at (A) 18°C and (B) 30°C.


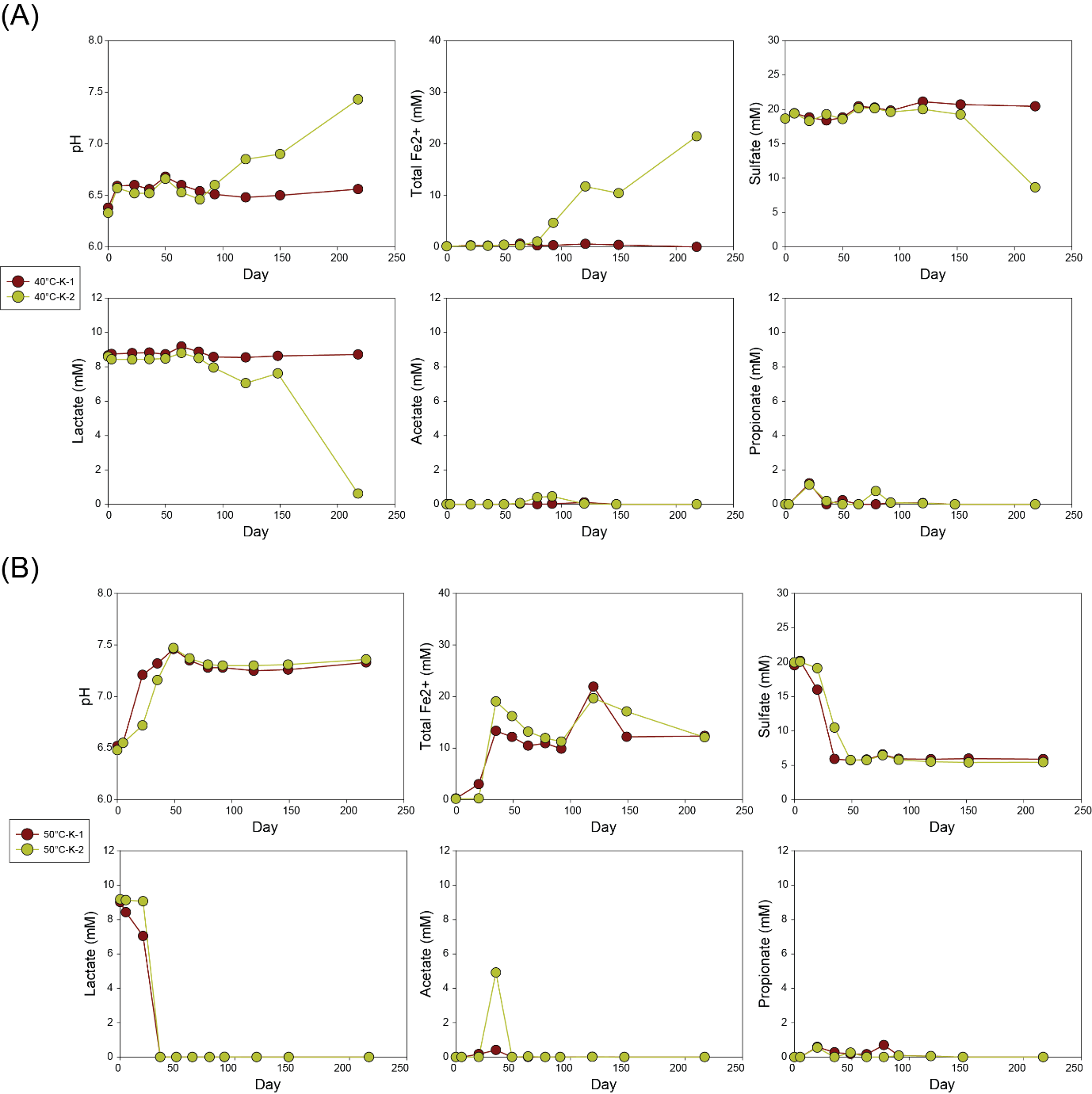


**Figure S2.** Changes in pH and concentrations of total Fe(II), sulfate, lactate, acetate, and propionate over time in kill control incubations at (B) 40°C and (C) 50°C.


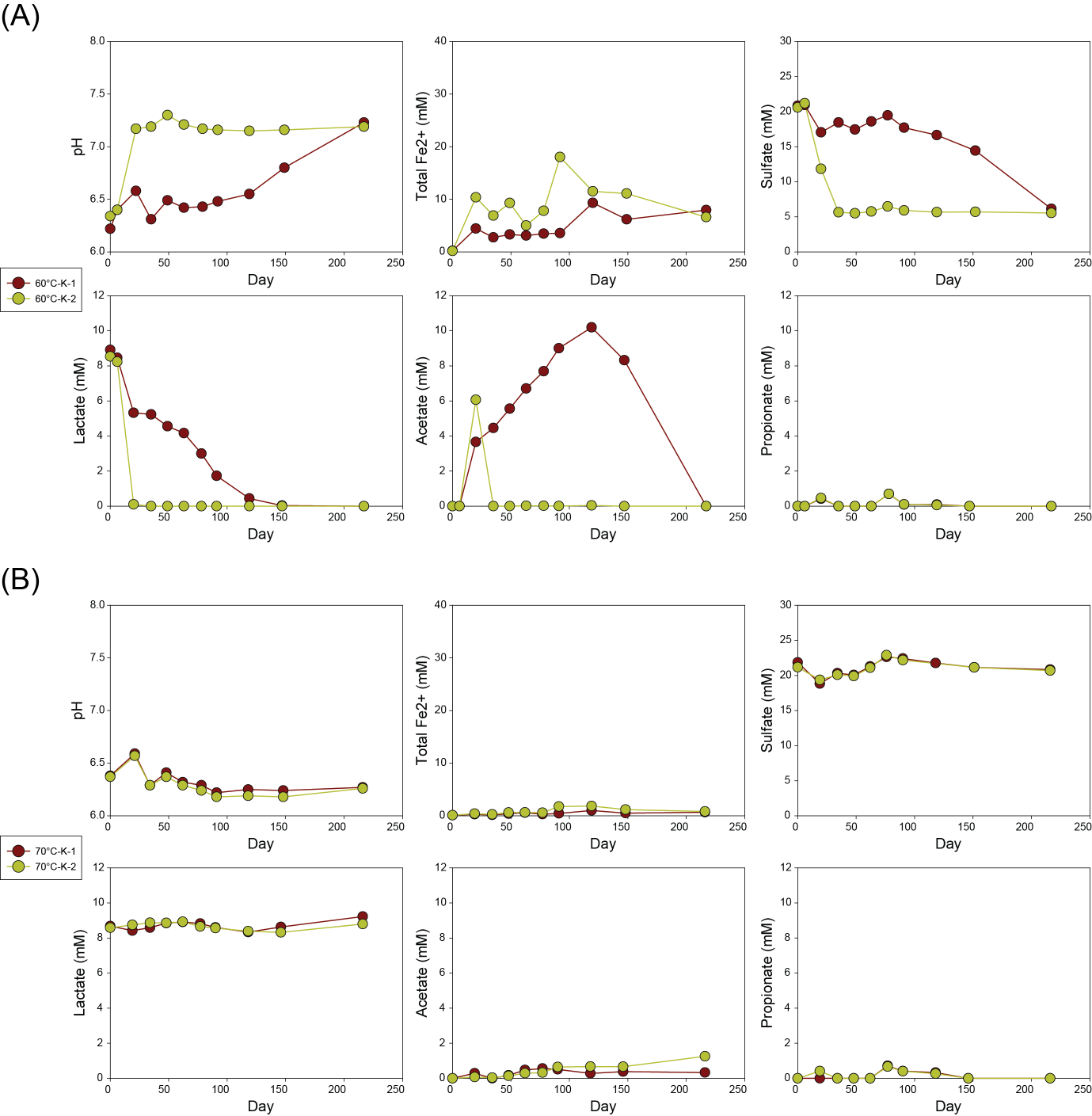


**Figure S3.** Changes in pH and concentrations of total Fe(II), sulfate, lactate, acetate, and propionate over time in kill control incubations at (A) 60°C and (B) 70°C.


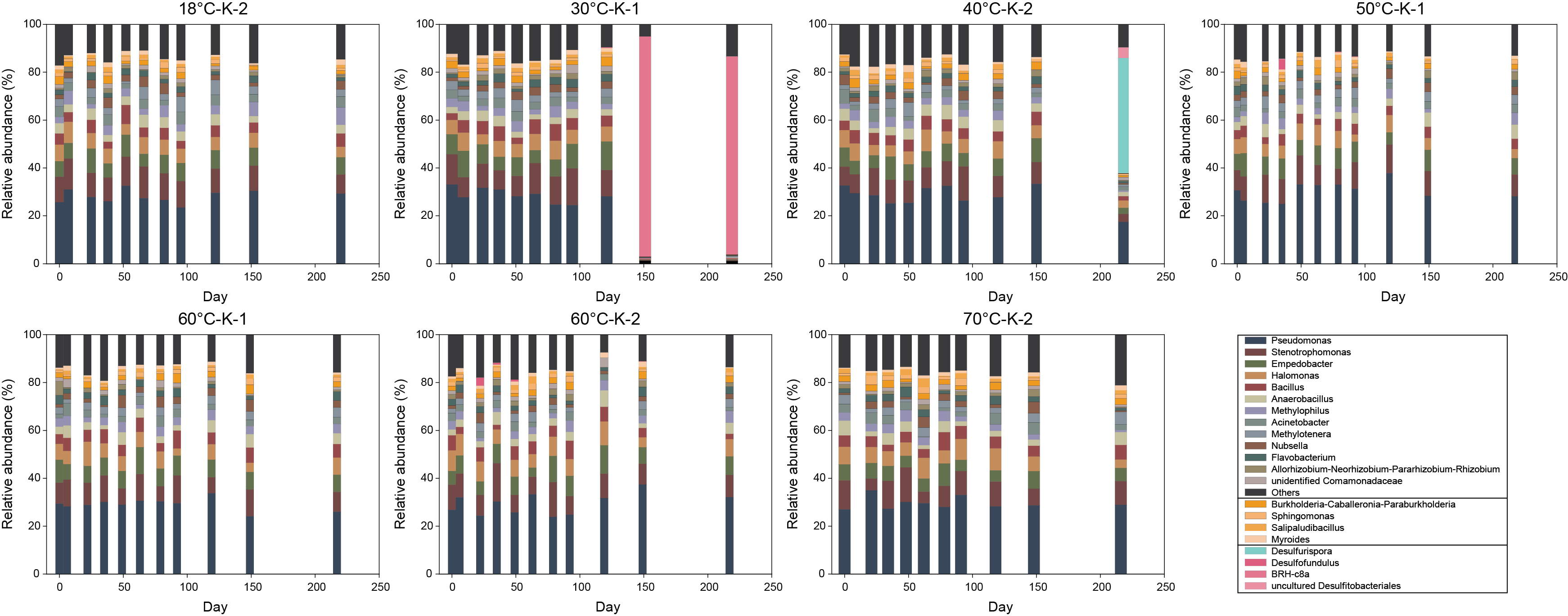


**Figure S4.** Microbial communities at genus level over time in kill control incubations

**References**

Park, S.-Y., Zhang, Y., O’Loughlin, E. J., Jo, H. Y., Kwon, J.-S., & Kwon, M. J. (2024). Temperature-dependent microbial reactions by indigenous microbes in bentonite under Fe(III)- and sulfate-reducing conditions. *Journal of Hazardous Materials, 465*, 133318. <https://doi.org/https://doi.org/10.1016/j.jhazmat.2023.133318>
